# Recent Advances in Multicellular Tumor Spheroid Generation for Drug Screening

**DOI:** 10.3390/bios11110445

**Published:** 2021-11-11

**Authors:** Kwang-Ho Lee, Tae-Hyung Kim

**Affiliations:** School of Integrative Engineering, Chung-Ang University, 84 Heukseuk-ro, Dongjak-gu, Seoul 06974, Korea; dlrhkdgh17@naver.com

**Keywords:** MCTs, ECM, drug screening, anticancer drug, 3D spheroid culture technology, culture platform, biosensing techniques

## Abstract

Multicellular tumor spheroids (MCTs) have been employed in biomedical fields owing to their advantage in designing a three-dimensional (3D) solid tumor model. For controlling multicellular cancer spheroids, mimicking the tumor extracellular matrix (ECM) microenvironment is important to understand cell–cell and cell–matrix interactions. In drug cytotoxicity assessments, MCTs provide better mimicry of conventional solid tumors that can precisely represent anticancer drug candidates’ effects. To generate incubate multicellular spheroids, researchers have developed several 3D multicellular spheroid culture technologies to establish a research background and a platform using tumor modelingvia advanced materials science, and biosensing techniques for drug-screening. In application, drug screening was performed in both invasive and non-invasive manners, according to their impact on the spheroids. Here, we review the trend of 3D spheroid culture technology and culture platforms, and their combination with various biosensing techniques for drug screening in the biomedical field.

## 1. Introduction

Cancer is currently widespread and is the cause of many deaths, regardless of gender and age. Unlike other diseases, cancer is believed to have a variety of causes, such as genetics, environmental factors, and acquired factors. Remarkably, factors that induce the formation of cancer are closely linked to intracellular interactions, creating an in vivo microenvironment. A tumor microenvironment (TME) consists of tumor cells; tumor stromal cells including stromal fibroblasts, endothelial cells, and microglia; immune cells such as macrophages and lymphocytes; and non-cellular components of the extracellular matrix such as collagen, fibronectin, hyaluronic acid, and laminin [1,2,3,4,5,6]. At the heart of the TME are tumor cells, which control the functions of both cellular and non-cellular components through complex signaling networks, using non-malignant signals to control them. As a result of this crosstalk, tumorigenesis and responses to multidrug resistance are linked [7,8]. Non-malignant cells in the TME are known to promote tumorigenesis at all stages of cancer development and metastasis [9,10,11]. In addition to the growth of the cancer, secondary tumors that develop in areas of the body that are far from the primary cancer are called “metastasis” [12,13,14]. The development of metastasis is a series of processes in which cancer cells leave the primary site, circulate through the bloodstream, withstand the pressure of blood vessels, and escape from combat with immune cells, i.e., as enemies, to the new cellular environment at the secondary site. Although metastasis is the major cause of cancer treatment failure and death, it is still poorly understood.

Researchers are currently studying cancer mechanisms and drug resistance using three-dimensional (3D) cell culture [15,16]. The existing two-dimensional (2D) culture system has enabled better understanding of complex cell physiology, and it can be used as the basis for biotechnology. However, 2D culture has a disadvantage that it cannot reflect cell-to-cell or cell-to-matrix interactions, and cell-matrix components that are important for differentiation, phagocytosis, and cell function in vivo [17,18,19]. Cell population produced through 3D culture systems exhibit characteristics that are more compatible with complex in vivo conditions [20]. Cultivation through 3D culture models not only leads to behaviors closer to natural conditions, but also exhibits novel and unexpected results on the tumorigenesis mechanisms [21]. The current trend is to utilize multi-cell culture rather than single-cell culture to simulate the microcellular environment and ensure cell interactions. Three-dimensional multicellular spheroids (MSCs) are gaining considerable attention in the biomedical field as they can simulate the interaction between cells and the environment of the extracellular matrix by emulating the structure and function of cellular tissues. In addition, multicellular tumor spheroids are used as 3D tumor models for anticancer drug screening due to their similar metabolic and proliferation gradient distribution to tumor tissue in vivo [22,23].

**Figure 1:**
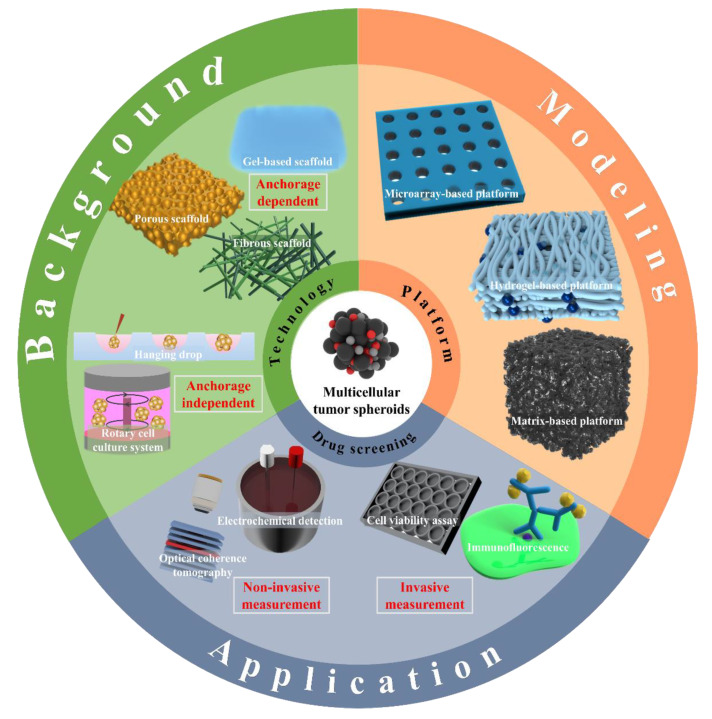
Multicellular tumor spheroid formation and drug screening through different approaches.

Therefore, multi-cultured cells in a 3D environment can serve as a cost-effective multi-drug screening platform for drug development and testing with in vivo mimicking models. This reduces the use of the existing animal clinical models and creates opportunities to evaluate the effects of drugs directly on humans. In terms of engineering, the configuration of a platform capable of realizing 3D cancer spheroids, appropriate cytotoxicity testing, and drug-screening methods should be considered in clinical cancer research [24]. In implementing 3D spheroids, factors such as appropriate material, cell adhesion strength, nutrient uptake, size, and extracellular matrix (ECM) components should be reflected, considering the TME. However, in terms of not harming 3D cells, difficulties persist in the process of cytotoxicity evaluation and drug screening using optical and electrochemical viability analysis kits and assays. In this review, a platform using a 3D multicellular spheroid was considered (Figure 1). First, the 3D cell culture technology was investigated to understand the technology being applied, and the series of processes in manufacturing the 3D co-culture spheroids using various methods was confirmed. In addition, the current trend of biosensing techniques was identified by discussing drug screening and cytotoxicity evaluation methods.

## 2. 3D Cell Culture Technology

Unlike the 2D cultures, which grow by attaching to the bottom as a monolayer, 3D cell culture refers to cells aggregated and expressed as a single tissue or form. Moreover, the 3D-cultured cells are attached to an artificially created ECM environment to interact with or grow with the surrounding environment. Therefore, unlike 2D cell cultures, cell growth in a 3D environment allows cells to grow in multiple directions rather than in a single direction in vitro, which is similar to in vivo conditions [25,26,27]. Upon comparison, the 3D cell culture exhibits several advantages: (1) A similar biomimetic model, which is more physiologically relevant. (2) A 3D culture exhibits a high level of structural complexity and maintains homeostasis for longer. (3) 3D models can indicate how different types of cells interact. (4) 3D cultures can reduce the use of animal models. (5) They are a good simulator for the treatment of disease groups including cancer tumors. The cell lines used in incubating multicellular spheroids are listed as below (Table 1).

### 2.1. Anchorage-Independent Approaches

Anchorage-independent 3D cell culture is a non-adherent cell culture system that is collectively referred to as a liquid-based system [45]. This system maintains cancer cells in a suspension to generate a self-assembly of tumor cells into a compact 3D aggregate known as a tumor spheroid or a cancer spheroid. Its main feature is that it enables the exchange of culture media to a certain point, although sophisticated handling is necessary due to the absence of an anchorage system to seize tumor spheroids. There are two pivotal liquid-based systems: (1) hanging-drop culture technology, (2) rotary-based culture system using spinner flasks and rotation culture system (RCCS) [46,47,48,49].

#### 2.1.1. Hanging-Drop Method

**Figure 2:**
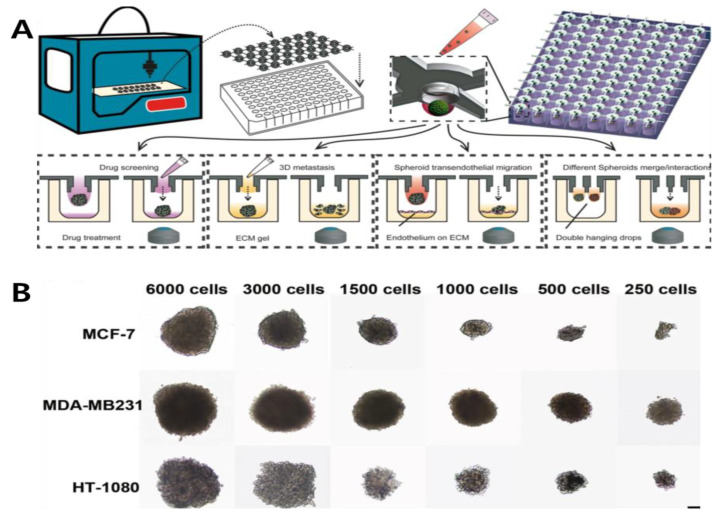
(**A**) Designing 3D-printed hanging-drop derivatives to investigate multicellular tumor spheroids. The device was printed for cancer spheroid formation on a 96/384 culture plate. Different types of assays are performed: drug screening, 3D metastasis, spheroid transendothelial migration, and spheroids merge/interactions. (**B**) Characterization of different cell lines cultured over two days with varying ratios. Reprinted with permission from [28]. Copyright 2019, Springer Nature.

MCTs can be easily formed using non-adhesive cell culture methods such as the hanging-drop method or centrifugation of cells in a suspension culture method [46,50], in which the cells in the suspension culture medium are located at the bottom due to the presence of a meniscus in the middle layer [51]. Conventional approaches to generate cancer cell aggregation are hindered by variations in the cell number and spheroid size, high-shear force, and their labor-intensive nature [52]. Recently, different microfabrication methods, including microfluidics and microwell, have exhibited the potential to form a large number of well-organized spheroids. Zhao et al., (2019) first introduced a 3D-printed hanging-drop dripper (3D-phd) device that enables long-term production of uniform cancer spheroids (Figure 2) [28]. In addition to culturing MCF-7 and MDA-MB-231 human breast cancer cells, this device performs a series of biomedical assessments, including imaging analysis, gene expression mapping, and anticancer drug assays. They proved that this device has some merits such as ease of design to perform 3D tumor migration analysis and can extend to organ-on-chip engineering, as well as assessing the anticancer drug efficiency on the co-culture spheroids. Some use the microfluidic technology, which is useful owing to its simple operation and enables point-of-care testing. Through this method, Park et al., (2020) devised a finger-actuated microfluidic device for spheroid cultivation and analysis that facilitates programmed media exchange and media injection for further analysis [29]. Different sizes of BT474 spheroids were generated after seven days of growing and further analyzed in a LIVE/DEAD assay, indicating its spheroid growth in a manipulated ECM-mimicking environment. The proposed microfluidic-based device can be widely applied in biomedical laboratories by combining it with automated machinery. For the formation of the MCTs, the manipulation of cell size uniformity and long-term cultivation is difficult to control. To overcome this limitation, thermoresponsive copolymers with a poly(N-isopropylacrylamide) (p(NIPA)) backbone were manufactured [30]. In this study, small-size spheroids of human breast adenocarcinoma cells were generated up to 2000 cells per drop. This demonstrated the superior performance of an in vivo 3D cell culture system. Through an immunofluorescence assay with the LIVE/DEAD analysis, these spheroids exhibited suitable drug penetration, making it a proper model for future drug-screening platforms. Hanging-drop is a conventional technology and has some limitations: (1) drops may fall off by mistake and (2) large amount of cells cannot be contained in one drop [53].

#### 2.1.2. Rotary Cell Culture System

The rotary cell culture system (RCCS) is a typical 3D cell culture system that involves both suspension and anchorage-independent cells. It is designed as a bioreactor system to simultaneously incorporate the ability to culture multiple types of cells with low turbulence and high mass transfer of nutrients. Jiang et al., (2019) investigated human MDA-MB-231 breast cancer cells in the RCCS to examine microgravity on the ultrastructure [31]. They aimed to determine the changes in the apoptosis, ultrastructure, and cycle progression for seven days. Through this trial, they pioneered new mechanisms and methods for preventing cancer cell metastasis and provided a deeper understanding of the treatment of malignant tumors. Recently, the RCCS bioreactor has been widely accepted as a microgravity simulation device. In one study, Chen et al., (2020) manipulated human HGC-27 gastric cancer cells cultured in an RCCS bioreactor system by simulating weightlessness [32]. Under this system, the effects of simulated microgravity (SMG) on the RCCS bioreactor were examined using liquid chromatography-mass spectrometry. Through this trial, the RCCS proved the importance of SMG, which has a major impact on lipid metabolism in cancer proliferation. This might be a novel target for treating gastric cancer disease. The RCCS enables uniform size and morphology of the spheroids. However, if the rotational speed is too high, the shear force becomes strong, which can affect the physiological response of the cells [54].

### 2.2. Anchorage-Independent Approaches

Mimicking the tumor microenvironment in culturing 3D cancer spheroids is the foremost consideration for guiding a successful 3D cell culture technology. Some studies utilize biomaterials to confine and attach cells three-dimensionally, such as encapsulating cells in hydrogels or growing cells in scaffolds [55,56,57,58]. Biomaterials are widely designed to facilitate cell adhesion, differentiation, and proliferation. They may comprise natural polymers, such as gelation, alginate, hyaluronic acid (HA), chitosan, and collagen or synthetic polymers, such as polycaprolactone (PCL), poly-L-lactic acid, poly (ethylene glycol) (PEG), polydimethylsiloxane (PMDS), and poly (lactic acid-co-caprolactone). A liquid mixture of the ECM can also be added directly to the culture media or numerous other ECM coating protocols can be employed to aid cell adhesion and stabilize cell aggregation to from 3D spheroids [59].

#### 2.2.1. Porous Scaffold

**Figure 3:**
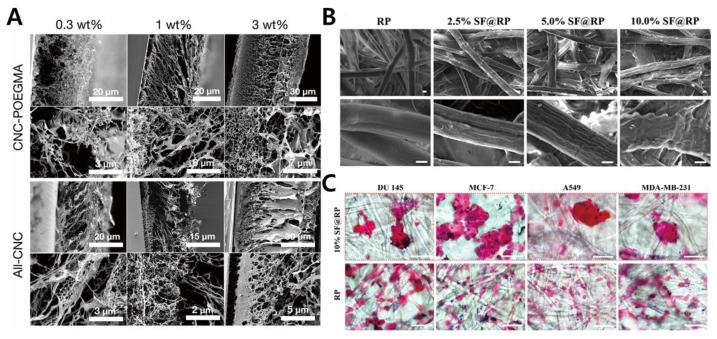
(**A**) Cross-sectional SEM images of the fabricated aerogel composite films with its porous 3D scaffolds. (**B**) FE-SEM microscopy of silk fibroin (SF)/rice paper (RP) composites with different concentrations indicating fibrous scaffolds. (**C**) Bright-field images of DU 145, MCF-7, A549, and MDA-MB-231 stained with H&E (red) in SF scaffolds. Reprinted with permission from [34]. Copyright 2019, American Chemical Society; Reprinted with permission from [35]. Copyright 2020, American Chemical Society.

A porous scaffold exhibits the following characteristics: porosity, pore size, morphology, which influences nutrient uptake for cell proliferation [60,61,62,63]. It has been widely accepted as a biodegradable polymer-based scaffold in tissue engineering; therefore, it needs to exhibit mechanical strength and flexibility [64]. Zhang et al., (2019) proposed a scaffold-based 3D cell culture system exploiting conductive polymer [33]. The importance of incorporating electroconductive material lies in its capability to sense electrochemical signals in a 3D cancer spheroid in a promising biocompatible polymer-based scaffold. A 3D porous PDMS scaffold was utilized to provide a favorable environment for a 3D cell culture. Then, a 3D PCP/Pt scaffold and PDMS scaffold were fabricated that exhibited stability and effectiveness in the 3D cell culture and tissue engineering. These scaffolds performed reactive oxygen species (ROS) monitoring of cancer cells, indicating great promise for future biomedical research. Different applications of the porous scaffold have been reported, which involved incorporating aerogel films and through pattering technology. Or et al., (2019) exhibited aerogel films with covalently cross-linked cellulose nanocrystals (CNCs) of designated dimensions and internal porous structures (Figure 3A) [34]. The usage of aerogel films has some merits in accelerating diffusion and reaction kinetics as a highly efficient catalyst matrix. Optimization of aerogel thickness and micropatterning were performed, and confocal imaging of human prostate cancer epithelial (PC3) cells showed its biocompatibility. In this work, they demonstrated an aerogel-based porous scaffold for a successful 3D cell culture technology.

#### 2.2.2. Fibrous Scaffold

Fibrous type scaffolds are an attractive biomaterial in tissue engineering owing to their ECM-mimicking structures, such as fibrous proteins, that exist in a native ECM [65,66]. Depending on the cell type, the fibrous scaffold can be manipulated to fabricate a suitable TME through physical attachments. In its universal application, fiber-based scaffolds can be fabricated into composite/hybrid scaffolds, microfluidic-based fibrous scaffolds, nanofibrous structures, or electrospun fibrous scaffolds [67,68]. Fu et al., (2020) developed silk fibroin (SF) scaffolds derived from silkworms in a fibrous protein in a cocoon. In this study, SF-coated rice paper (RP) was fabricated, and it was prepared through a one-step dip-coating protocol (Figure 3B,C) [35]. To prove its biocompatibility, human breast, lung, and liver cancer cells were cultured on this platform, successfully demonstrating spheroid formation. To confirm its cell viability assessment, MTT assay and cell staining were performed, each demonstrating its potential for large-scale clinical application. Drug sensitivity was also investigated.

#### 2.2.3. Gel-Based Scaffolds

Gel-based scaffolds can be modeled directly into an unstructured molded tissue. However, their stability is weak; therefore, they can be used with tissues under load such as bones. Biomaterials for gel-type 3D cell culture are derived from natural and artificially modified materials [69]. Since gels allow independent control of matrix and ECM functionalization, Ashworth et al., (2020) demonstrated a self-assembling peptide gel for designing the 3D cell culture [70]. By controlling stiffness through peptide concentration and pH condition, the peptide gel was fully utilized with further experiments on culturing breast cancer. An analysis of the results obtained through immunofluorescence staining and quantitative reverse transcription polymerase chain reaction (qRT-PCR) indicated that the peptide gel can be used to model the progression of breast cancer, independent of the matrix microenvironment regulation. Some researchers reported a self-assembling peptide, bQ13, that can be useful for obtaining the 3D culture of prostate cancer cells [36]. These self-assembled peptides have been known to help stabilize 3D culture and provide a user-defined matrix that can be tailored with different experimental conditions [71]. Investigation of the rheological properties of the peptides proved its maintenance in an ungelled state at a basic pH. Additional examinations of cell encapsulation and survival through immunostaining showed a well-organized, non-polarized morphology within the prostate spheroids, indicating an attractive scaffold for further modification. Hence, hydrogel scaffolds allow minimal damage to the spheroids and facilitate transport of nutrients and water as compared to other porous and fibrous scaffolds.

## 3. Multicellular Cancer Spheroid Formation Platform

Through 3D cell culture technologies, 3D cell culture platforms have been developed to recapitulate an in vivo microenvironment of solid tumors. Mimicking physiological characteristics of the TMEs, such as tumor metastasis, angiogenesis, and tumor-stromal interaction, enables researchers to achieve a cellular behavior closer to natural conditions [72,73,74]. Further, 3D cancer spheroid models have received wide attention for their potential applications in drug-screening assessments. Notably, 3D multicellular tumor spheroids can be used in the investigation of the TME regulation of tumor physiology and therapeutic obstacles associated with the proliferative and metabolic gradients in a 3D spheroid context [75]. In molecular biology approaches, the genomic stability of multicellular spheroids is superior to that observed in a 2D monolayer culture in terms of gene expression, DNA, and RNA level [76]. Since the MCTs can exhibit sophisticated in vivo solid tumor behaviors, researchers utilized a clinical drug-screening tool based on the 3D MCTs formation platform.

### 3.1. Hydrogel-Based Platforms

**Figure 4:**
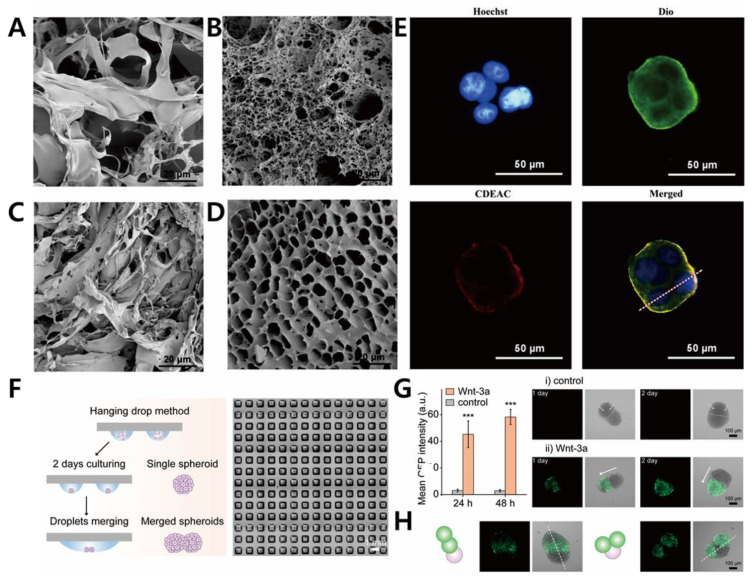
Morphology of the hydrogel platform after freeze-drying, investigated using scanning electron microscopy (SEM), with the SEM images of Eu (III) complex and carboxymethyl cellulose (CMC) backbone (**A**), the formed colloidally stable suspensions (CDEAC) (**B**), ClO^−^-added aqueous solution (**C**), and recovered state of the hydrogel upon addition of SCN^−^ (**D**). (**E**) Multicellular cancer spheroids of MCF-7 from the CDEAC hydrogel stained with Hoechst (Blue), Dio (green), and CDEAC hydrogel (red). (**F**) Culture mechanism of the programmable assembly of spheroids with hydrophobic borders. (**G**) Calculated activation intensity of the multicellular complex of Wnt-3a and GFP-labeled HEK spheroids. (Intensity of GFP was estimated from at least 10 spheroids. ***, *p* < 0.001, one-way ANOVA). (**H**) Fluorescence microscopy of triple spheroids with Wnt producer spheroid and Wnt reporter spheroids. Reprinted with permission from [38]. Copyright 2019, Elsevier; reprinted with permission from [42]. Copyright 2020, Wiley Online Library.

Hydrogels are widely studied for bioengineering applications, such as regenerative tissue engineering, 3D cell culture, and drug delivery, due to their ECM-mimicking structures [66,77,78,79,80,81]. The hydrogels exhibit 3D hydrophilic networks that show high water content and are advantageous in transporting nutrients, oxygen, and other water-soluble metabolites. In the formation of MCTs, an aqueous droplet or gel solution encompassing spheroids are guided to reduce the number of preparation steps such as culturing cells, maintenance, controlling cell aggregation, and delivery of reagents [82,83,84]. Recently, numerous acoustofluidic devices have been devised to load a single type of cell into aqueous droplets or to generate MTS assemblies of microchannels, an aqueous two-phase system (ATPS) [85,86]. ATPSs allow simplified culture preparation, maintenance of cancer cells, and aggregation of MCTs. In this way, Chen, Bin et al., (2019) developed a microfluidic platform for synthesizing dextran/alginate (DEX/ALG) hydrogel spheres that enable templated fabrication of multicellular spheroids in an ATPS [37]. The principle of fabricated acoustofluidic device lies in the following: (1) PEG- and DEX-enriched phases including the gel-forming agent ALG are pumped into the device. (2) Amplified acoustic flow stream is applied to the inner fluid with frequency control. (3) Droplets are cross-linked in a calcium bath to fabricate a hydrogel and transferred to the suspension culture system where the MTSs are formed. In order to culture mouse mammary carcinoma (EMT6) multicellular spheroids for a lengthy duration, this platform ensured long-term cultivation, uniform multicellular spheroids, and suspension culture conditions with growth factors to enable further analysis of organoid development. However, using enzymes or light ligands on the hydrogels is reported to be harmful to spheroid integrity [87,88]. To overcome these issues, researchers implemented anions that are reversibly responsive luminescent nanocellulose hydrogels for efficient formation and release of multicellular spheroids (Figure 4A–E) [38]. By mixing Eu(III) complex laden carboxymethyl cellulose (Eu(III) complex-CMC) and 2,6-pyridinedicarboxylic acid functionalized CMC (K-DPY-CMC), efficient regulation of hydrogel formation and release of entrapped MCF-7 breast cancer spheroids was accessible. The proposed hydrogels have a class of ClO^−^/SCN^−^ reversibly responsive anions with fluorescence activation/deactivation. First, addition of a ClO^−^-induced destruction of the nanocellulose hydrogel network, accompanying fluorescent quenching. Upon addition of SCN^−^, fluorescence hydrogel was recovered by cross-linking of ClO^−^/SCN^−^ with precise regulation. Further, the 2,5-diphenyl-2H-tetrazolium bromide (MTT)x assay was performed to test in vitro cytotoxicity. Additionally, Fourier-transform infrared spectroscopy (FT-IR), scanning electron microscopy (SEM), and a rheometer were implemented to optimize the concentration of the hydrogel. Upon formation of MCF-7 multicellular spheroids (human breast cancer cells), it could be easily released through ClO^−^/SCN^−^ regulation and monitored in a time-dependent manner through fluorescence imaging. However, there still exist some problems associated with the incomplete adherence of the spheroid on the substrate and difficulties in regulating the cellular movement and cell density on the outer membrane of the encapsulated cells [89]. Ramadhan et al., (2020) suggested a redox-responsive hydrogel that enables self-wrapping co-culture [39]. Its mechanism involves decomposition of hydrogels under mild reductive microenvironments, and the peeling-off of a monolayer of cells cultured on a redox-responsive hydrogel surface that self-folds to wrap other cell lines. The decomposition of a redox-degradable PEG-based hydrogel can be controlled via the concentration of cysteine (CYs), indicating that the detachment of cell membrane can be regulated. Optimization of the self-folding process of a fibroblast cell line (NIH3T3) to warp liver hepatocellular carcinoma (HepG2) and human umbilical vein endothelial cells (HUVECs) into higher-order microstructures. Since the ECM component is crucial for 3D tumor formation, a collagen bead was added and the result was remarkably favorable in that the size of the tumor increased gradually and the necrotic area was diminished dramatically, indicating its enhanced spheroid cell viability. 

There still exist some issues with controlling cell aggregation and growth of multicellular spheroid. However, there are advantages that offset those challenges in terms of its diverse usage, biocompatibility, user-friendly suspension culture platform.

### 3.2. Microarray-Based Platform

Cells cultured in a micro-patterned array format are attracting tremendous attention in screening drug candidates for toxicity and efficacy in clinical trials [90]. Recent studies have proved that an in vitro microarray culture platform is an effective drug-screening tool with reduced cost and time that dramatically reduces the need to perform animal tests [91,92]. Moreover, 3D microarray culture platforms enable spheroid analysis in terms of drug treatment response, cell–extracellular matrix, and cell–cell interactions in a high-throughput manner. Thus, 3D microarrays provide an excellent alternative to conventional 2D plate-based assays [93,94,95]. Several methods have been developed to fabricate 3D microarray platforms including surface patterning, soft lithography, cell printing, and microfluidic-based application. The spatial position and morphology of the microarray is important for successful cell aggregation, and for multicellular spheroids to form stable co-cultures of multiple types of cells. To overcome these technical obstacles, different controlled arrangements of the microarray-based 3D spheroid culturing method have been reported. 

The application of CO_2_ laser ablation in the fabrication of the microarray has provided a considerably rapid and economical technique for generating multicellular spheroids utilizing size-controlled microwells [96,97,98]. Wu et al., (2021) published a reproducible U-shaped microwell array that facilitates high-throughput 3D tumor spheroid culture [40]. The size of the microwells is considered for precise manipulation of horizontal spacing (d_x_) and vertical spacing (d_y_) of an array for preventing cell loss during cell seeding. A549, Huh-7, and T24 multicellular spheroids were cultured in different sizes of the microwell, indicating the importance of optimization of the microwell size and seeding cell amount from different cell lines. Following this method, a size-controlled MCTs microarray culture platform was designed as an in vitro tumor-mimicking model to probe the drug-screening target. Microwells arrays on hydrogel assays form an interesting research topic as the 3D architecture of the array can be controlled. Dhamecha et al., (2021) suggested thermoresponsive hydrogel microwell array platforms that facilitate stress-free generation and isolation of the multicellular cancer spheroids [41]. In this assay, the poly N-isopropylacrylamide-based hydrogel microwell array (PHMA) was used, enabling the growth and aggregation of spheroids at 37 °C and convenient isolation of spheroids at room temperature (25 °C). A549, HeLa, and MG-63 cancer cell lines with human lung fibroblasts (HLF) were incubated in PHMA, forming multicellular spheroids with a spherical morphology with hypoxic cores. The swelling and de-swelling behavior of the PHMA allowed detachment of spheroids at room temperature, indicating its potential as a disease modeling platform and for drug-screening assessments. Cui et al., (2021) used the droplet-fusion technique to construct various multicellular structures in a miniaturized high-density assay format (Figure 4F–H) [42]. The droplet microarray (DMA) platform enables production of nanoliter droplet microarrays where the size, shape, and density of droplets depend on the style of the hydrophilic patterns surrounded by the hydrophobic barriers. The designed platform can cultivate and screen various types of cells in individual nanoliter droplets as miniaturized TMEs [99]. Through the modulation of the size and distance between the hydrophilic spots on the DMA, PROgrammable Merging of Adjacent Droplets (proMAD) can be employed to generate 3D multicellular spheroids by fusing multiple neighboring droplets of single spheroids. Thus, the proMAD method can be applied to the pharmacokinetic field where high-throughput screening and generation of hetero-type spheroids are required. Currently, graphene and its derivatives have shown promise in improving cell adhesion due to rapid absorption of ECM materials [100,101]. Namely, Kim et al., (2020) developed a graphene-oxide (GO) microarray platform that exhibited efficient cancer spheroid formation [102]. The HepG2 cells were cultured on this vertically coated GO platform, forming spheroids that grew from outside to inside. By treating with different anticancer drugs, the spheroid sizes could be quantitively monitored at various concentrations of the drugs. 

Recently, researchers envisioned a novel platform to guide facile and highly reproducible fabrication method to reliably generate MCTs. In numerous attempts to overcome unwanted irregular spheroid growth, a customized microarray-based platform has been fabricated, with its size and shape well-suited to multicellular spheroid growth.

### 3.3. Matrix-Based Platform

**Figure 5:**
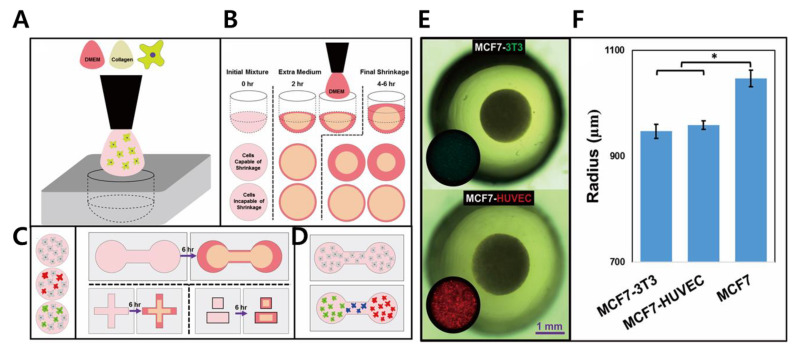
(**A**) Fabrication process of the collagen matrix platform. (**B**) Cell consolidation and shrinkage due to structural transformation and medium addition. (**C**) Creation of homogeneous co-culture structure with different morphologies (dumbbells, cuboids, and crosses). (**D**) Heterogeneous components formed by different cells at specific location of the molds. (**E**) Bright field and fluorescence microscopy of MCF-3T3 (green) and HUVEC (red) co-cultured with MCF7. (**F**) Calculated radius of a spheroid depending on cell type. (*p*-values: * < 0.01). Reprinted with permission from [43]. Copyright 2019, Elsevier.

In the regenerative and tissue engineering field, bio-mimicking scaffolds are routinely used to provide mechanical support for cell growth and tissue repair [103,104]. Biomaterials are derived from both natural materials and synthetic polymers with biocompatible properties [105]. Some synthetic polymers and polysaccharides such as HA, chitosan, alginate, poly(lactic-co-glycolic acid), and PEG have excellent physicochemical abilities and can be fabricated with minimum variability [106,107]. To engineer tumor ECMs suitable for cell adhesion, these biomaterials require further modification with integrin-binding domains to guarantee MCT formation [108]. In contrast to synthetic polymers, natural polymers such as collagen and Matrigel have been used in numerous approaches owing to their inherent cytocompatibility [109]. Earlier versions of scaffolds were limited to low cell numbers with slow processes and specific type of cells. At present, versatile techniques for handling scaffolds with diverse shapes and various types of cells co-cultured or compatible with tumor cell microenvironment have been developed [110]. In a previous study, a platform with rapid self-assembly of cells and matrix material of various shapes using microfabricated molds was introduced (Figure 5) [43]. Researchers have demonstrated that various molds with dumbbell, cross-like, spherical, and cuboidal shaped cell morphologies could be generated on this platform. Using this approach, different cell lines, such as breast cancer, osteosarcoma, and endothelial cell lines, were cultured with a range of cell seeding amounts. Non-spherical molds like dumbbell and cuboidal shapes retained their shape even after elimination from the molds and during long periods of culturing. Additionally, the shape of molds could be patterned to position numerous cell types in an accurate and controlled way, indicating a significant role in tissue or organ implantation. 

Matrix-based scaffolds could also be applied to multiple biomaterials to better mimic a 3D in-vivo tumor. For instance, polyurethane (PU) was used in the scaffolds with enhanced long-term multicellular incubation involving cancer and endothelial cells [44]. The purpose of the study was to mimic pancreatic ductal adenocarcinoma (PDAC), which is a deadly disease. Two different compartments were organized: an inner tumor zone coated with fibronectin (FN) for cancer cell growth and a surrounding stromal zone treated with collagen I (COL) facilitating stellate and endothelial cell adhesion. Three types of cells were successfully generated in vitro with the shape of the scaffolds varying according to their usage and target cell lines. Zhang et al., (2020) illustrated cross-linked nanofibrous-type scaffolds with the usage of alkaline phosphatase (ALP) and carboxylesterase (CES) [111]. Two biomaterials were guided in a designated manner to induce coexistence of nanofibrils and vesicles, followed by the generation of nanoaggregates, resulting in the cross-linked scaffolds. 

Overall, synthetic polymers have certain limitations in recapitulating precise structure and composition of ECM, which leads to inflammatory responses induced by the implantation of the fabricated materials. Native biopolymers derived from ECM have excellent biocompatibility and generally facilitate regenerative response after engraftment. However, they have limitations such that the recreation of the nanostructure of native ECM using single or multiple biopolymers is difficult. 

## 4. Biosensing Methods to Assess Drug Efficacy in Multicellular Spheroids

Multicellular spheroids are used in drug screening tools as an in-vitro spheroid model for the selection and identification of drug candidates [112]. Because MCTs are superior to other 2D culture cells in producing physiological conditions of tumors, such as oxygen mobility, nutrients, and drugs, they have been subjected to a more sophisticated model of in-vivo drug testing assessments. Furthermore, drug delivery could be administered to MCTs models with a facile clinical evaluation [113,114]. Since drug uptake and diffusion were accurately replicated in 3D multicellular models, MCTs could be applied to drug penetration analysis. Different approaches have been developed to assess drug-screening platforms, such as immunofluorescence, fluorescence activated cell sorter (FACS), absorbance assay, electrochemical detection (ECD), and optical coherence tomography. In this study, the abovementioned tools are classified into two types: invasive and non-invasive measurements, depending on the characteristics of each measurement. In this section, various biosensing tools that have been used to assess the efficacy of drugs in multicellular cancer spheroids are discussed (Table 2).

### 4.1. Invasive Sensing Methods

In invasive screening, conventional analyses have been used for drug-screening measurements to evaluate drug toxicity. For instance, invasive screening could be introduced to MCTs monitoring in two approaches: immunofluorescence and cell availability assays. Inevitably, damage arises in these drug-screening assessments, influencing the overall results of drug-screening experiments. Additionally, invasive screening necessitates fixation of cells, addition of toxic reagents, and the breakdown of cell populations into separate cells. 

#### 4.1.1. Immunofluorescence

**Figure 6:**
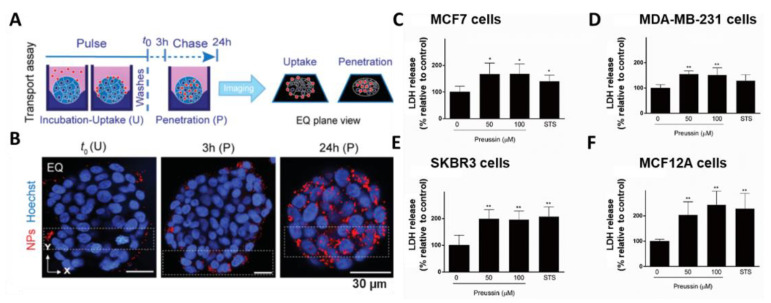
(**A**) Schematic illustration of transport assay for sensing NP drug uptake and penetration. (**B**) Confocal microscopic images showing spheroids at different stages of the NP uptake wave at Z-stacks. LDH cell viability assay for evaluating cytotoxic effect of preussin at different concentrations of 0 µM, 50 µM, 100 µM, and STS (1 µM) on (**C**) MCF7, (**D**) MDA-MB-231, (**E**) SKBR3, and (**F**) MCF-12A cells. (* *p* < 0.05; ** *p* < 0.01). Reprinted with permission from [117]. Copyright 2019, Wiley Online Library; Reprinted with permission from [119]. Copyright 2019, MDPI.

Immunofluorescence is a worldwide tool that facilitates vivid and cellular monitoring in biological studies [124,125]. The monitoring mechanism involves using specific antibodies that are chemically compatible with fluorescent dyes. Once conjugated to antigen–dye complexes, these labeled antibodies bind to cellular antigens, which can be visualized in a fluorescence image. This technique is useful to demonstrate target antigens in tissues or circulating fluids, assisting diagnosis and monitoring of life-threatening disorders. Because cancers are chronic diseases worldwide, several studies using an immunofluorescence assay have been reported. In a previous study, an acoustic droplet-based microarray platform was engineered to facilitate screening of patient-derived spheroids [115]. For rapid and precise screening of cultivated human samples, MCF-7, HeLa, and Caco-2 cells were stained with calcein and PI. Additionally, fluorescence 3D modeling was performed for clinical demonstration of patient-derived spheroids. Through immunofluorescence analysis, protein expression was visually provided comparing before and after drug treatments. In drug-screening analysis, the drug penetration assessment is an indispensable component to confirm the efficacy of anticancer drugs. Machálková et al., (2019) performed drug penetration analysis on spheroids by laser scanning confocal microscopy and matrix-assisted laser desorption/ionization mass spectrometry imaging [116]. Human colorectal carcinoma (HT-29) cells were cultured, forming multicellular spheroids. Thereafter, the cells were treated with perifosine drug. d, drug distribution was confirmed, and the spheroid regions were colocalized with apoptosis, proliferation, and metastatic features. In modeling NP penetration for drug delivery, it is necessary to mimic tumor microenvironments, allowing specific interactions between the ligands on the surface of carrier and tumor cells [66]. Cutrona et al., (2019) demonstrated uptake and transport of NPs in spheroids using an automated confocal microscopy (Figure 6A,B) [117]. Profiling HT-29 multicellular spheroids, quantitative analysis was performed to observe penetration of synthetic NPs. The morphology of 4 days of HT-29 spheroids was observed through β-catenin, TGN46, actin, and nuclei staining, followed by Z-stack confocal imaging. Above all, the penetration studies of drug screening illustrated therapeutic target across tumor cells in vivo. Immunofluorescence analysis allows fast monitoring and precise quantification for drug screening at cellular and molecular levels. However, immunofluorescence assays have limitations such as staining compact spheroids with a dye unable to penetrate to the inner cells. Basically, staining protocols necessitate fixation steps that results in unavoidable death of target spheroids.

#### 4.1.2. Cell Viability Assay

When cancer cells proliferate or die, they emit specific biomarkers [126,127,128]. In drug screening, cells treated with toxic compounds undergo two phenomena: proliferation stops or necrosis arises, and apoptosis, which leads to cell death [129]. Basically, necrosis refers to swelling cells and bursting membranes emitting inner cellular components, caused by toxic chemicals or sudden physical stress [130]. In case of apoptosis, cells contract, DNA breaks down into specific fragments, and cells eventually die after a series of processes such as breakdown by blood cells [131]. Various cell-based assays, such as tetrazolium reduction assays (MTT, MTS, XTT, and WST-1), lactate dehydrogenase assay (LDH), Cell Counting Kit-8 (CCK-8), hematoxylin and eosin (H&E) assay, and terminal deoxynucleotidyl-transferase-mediated dUTP nick-end labeling apoptosis assays [132,133,134,135,136,137], have been reported. Fundamentally, cell viability assays are based on detecting cellular components using reagents, dyes, and a series of reduction-mediating electrons. All of cell viability assays require incubation of a proper reagent with an estimated number of viable cells to label fluorescent byproducts that can be visualized through a plate reader. In one study, anticancer activity of copper (II)–tropolone complex was investigated in breast multicellular spheroids [118]. MCF7 (breast adenocarcinoma) and MDA-MB-231 (triple-negative breast adenocarcinoma) multicellular spheroids were generated, and a cytotoxicity assay, the MTT assay, was performed to determine the anticancer effect of Cu(trp)_2_ in comparison with the conventional drugs of cisplatin (CDDP) and doxorubicin. The IC_50_ values of Cu(trp)_2_ were four- and sevenfold lower than IC_50_ values of CDDP on MCF7 and MDA-MB-231 cells, indicating the anticancer effect of Cu(trp)_2_. LDH assays have also been used in breast cancer studies profiling a cytotoxic effect of preussin drug derived from the marine-sponge-associated fungus (Figure 6C–F) [119]. The anticancer effect of preussin at concentrations of 50 and 100 μM on MCF7, SKBR3, and MCF12A cells resulted in approximately 100% LDH release compared to control groups. Since LDH released from damaging cells, the amount of LDH release correlated to drug efficacy of preussin. In addition, Murphy et al., (2021) suggested a drug delivery model incorporating with manganese dioxide nanoparticles (MnO_2_ NPs) to design controlled oxygen production and promote nature killer cell function [120]. To increase biocompatibility, MnO2 nanoparticles were encapsulated to poly(lactic-co-glytic), forming PLGA-MnO_2_ NPs. The cytotoxicity of proposed NPs was analyzed using the MTS assay on MCF-7 multicellular spheroids. In this assay, PLGA-MnO2 NPs exhibited significantly enhanced biocompatibility compared to PEG-MnO2 NPs. In diverse approaches, cell viability assays successfully quantified drug efficacy in a detailed and precise manner. 

### 4.2. Non-Invasive Sensing Methods

Recently, cytotoxic assessments of drug candidates in a non-destructive manner were designed to exhibit precise drug screening results [138]. Non-invasive measures should be cell-friendly and non-toxic to cells, making them ideal for determining anticancer drugs’ effects. Likewise, measuring spheroid viability via non-invasive screening has been considered as an alternative to conventional colorimetric assays. In general, non-invasive methods do not affect the internal or external cellular environment and allow normal physiological metabolism; thus, it is possible to monitor and evaluate the original appearance of the cell. Ultimately, methods that omit the destructive pretreatment steps can be an excellent index that can clearly reflect the cells in the living organism.

#### 4.2.1. Electrochemical Biosensing

**Figure 7:**
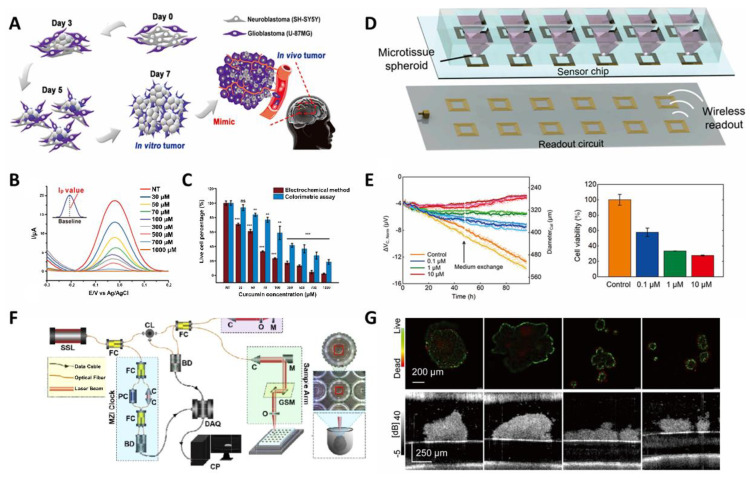
(**A**) Schematic diagram of a real tumor-mimicking Co-culture spheroid model. (**B**) DPV signal graph of the brain tumor model treated with varying concentrations of curcumin. (**C**) Calculated live cell percentage derived from the electrochemical signal peak (Ip_a_) from (**B**) and CCK-8 results indicated in a bar graph. (Student’s *t*-test, *N* = 3, * *p* < 0.05, ** *p* < 0.01, *** *p* < 0.001). (**D**) An overview of the wireless impedance-based sensor platform that was connected to a PDMS microfluidic device. (**E**) Normalized ΔVc,_Norm_ values of 0.1, 1, and 10 μM doxorubicin samples and standard medium conditions (control). ΔV_C_ indicates evaporation-compensated voltage shift. (**F**) Validated ATP measurements of cancer spheroids after 96 h of incubation. The cell viability values were based on the control samples (the error bar indicating mean absolute errors, *N* = 2). (**F**) Schematic of the OCT system for 3D imaging of the spheroid. (**G**) Cross-sectional drug toxicity evaluation of the MCF7 spheroids. The upper panel shows the fluorescence images and the lower panel shows the results obtained through the OCT intensity microscopy. Reprinted with permission from [122]. Copyright 2020, Wiley Online Library; Reprinted with permission from [121]. Copyright 2020, American Chemical Society; Reprinted with permission from [146]. Copyright 2021, Optical Society of America; Reprinted with permission from [110]. Copyright 2020, Optical Society of America.

Recently, an ECD method with a fast and sensitive technique was considered as a drug-screening tool [139,140,141]. When an ECD system is electrically stimulated, the chemical response of the stimulus can be observed, which measures an analyte electrochemically. The electrochemical reaction involves oxidation/reduction via movement of electrons. Depending on the type of electrical signals and regulation factors, various electrochemical measurements exist, such as linear sweep voltammetry, cyclic voltammetry, differential pulse voltammetry (DPV), chronoamperometry, chronopotentiometry, electrochemical impedance spectroscopy (EIS), and electrical impedance tomography. The electrochemical signal intensity is proportional to the cell population, indicating cell viability. Dong et al., (2020) demonstrated monitoring of MCTs in a parallelized wireless sensing system (Figure 7D–F) [121]. An EIS-based platform was devised to continuously measure the size and viability of cancer microtissues for 90 h. The cancer microtissues were treated with different concentrations of doxorubicin (0.1, 1, and 10 μM), which is widely known as a chemotherapeutic agent. The diameter and cell viability were decreased with increasing concentration of doxorubicin in HCT-116. Some researchers have applied the hybrid function platform to MCTs by increasing the electrochemical signal intensity. In one study, a gold-nanostructure-based platform was designed to detect curcumin, which is known as a natural anticancer compound in a brain cancer model (Figure 7A–C) [122]. On this platform, a neuroblastoma (SH-SY5Y) and glioblastoma (U87-MG) were co-cultured to form spontaneous multicellular spheroids without any treatment. Comparing the toxicity assessments of DPV and CCK-8, the electrochemical method proved to be more sensitive (29.4%) with a low concentration of curcumin (30 μM). Spheroids were also monitored for a long time, enabling real-time, non-invasive analysis of potential drug candidates. 

Electrochemical detection analysis proved its precision and rapidness without damaging the cellular components, making it an excellent method for monitoring spheroid viability. Preparation steps are minimized such that only the electrochemical device and a live sample are required for the analysis. Above all, live multicellular spheroids can be preserved before and after electrochemical detection. Since the redox reaction is a sudden response, optimization of external components is required.

#### 4.2.2. Optical Coherence Tomography-Based Biosensing

Optical coherence tomography (OCT) refers to a technique capable of imaging an entire spheroid with a high resolution and millimeter-scale penetration depth, in addition to providing physiological and morphological cues about the spheroid [142,143]. It allows label-free imaging, using the intrinsic contrast lens to analyze a sample in a non-destructive manner, thereby enabling clinical cancer research. The OCT technology can be used to analyze the pharmacokinetic response of drug candidates and can be applied to determine the microenvironment and vascular interactions of tumors [144,145]. OCT provides tissue morphology at much higher resolutions than other imaging models such as MRI and ultrasound. The impermeable region of inner spheroid biology can be investigated through the OCT analysis. Confocal microscopy can be used to examine multicellular spheroids through greater depth of imaging. Hari et al., (2019) used this method to conduct refractive index measurements of human colon cancer (HCT116) spheroids [123]. The aim of this research was to study the presence of hypoxia in oxygen-poor cores of the spheroids, which have been known to play an important role in tumor proliferation. The core of the HCT116 spheroids showed debris of stained cell components after sufficient maturity. This study demonstrated the spheroid imaging of hypoxia that could be a future target for the drug-screening analysis. In other cell lines, ovarian cancer cell (OVCAR-8) was applied to visualize 3D structures and monitor necrotic zones within the MCTs (Figure 7F) [146]. In this study, a swept-source optical coherence tomography platform was fabricated, and OCT images were obtained. Mathematical models were employed to calculate growth kinetics of the spheroid size and necrotic tissues. The root-mean-square error (d)used an indicator to evaluate mathematical models, and lower scores showed better performance. Remarkably, the Boltzmann model proved facile performance as it had the lowest RMSE and AIC score for the growth kinetics of multicellular ovarian tumor spheroids. This result demonstrated OCT for potential drug-screening development for cancer research. Recently, the OCT has been employed not only to determine the tissue structure, but also tissue dynamics. It can be used to assess the necrotic tissue dynamics in multicellular spheroids, which is crucial to investigate the drug efficacy response [147,148]. Two OCT-based methods were reported, such as logarithmic intensity variance (LIV) and OCT correlation decay speed analysis (OCDS), a technique that quantifies and visualizes tissue dynamics (Figure 7G) [110]. Further, human breast adenocarcinoma (MCF7) multicellular spheroids were visualized using OCT, and two methods were used to evaluate the tissue dynamics. First, the LIV methods indicated that necrosis occurred at the center due to lack of nutrient uptake, which is consistent with the necrotic process of a solid tumor. The gradual increase in the OCDS plots demonstrated that the necrotic response became more intense. After paclitaxel drug treatment, the indicators of the necrotic process occurred at the outer region of the spheroids, which was investigated through following analysis. 

Overall, OCT allows non-invasive and in-depth analysis tumor imaging. However, some limitations exist in that measurable depth is limited and application samples are restricted. 

## 5. Conclusions

Through the studies discussed above, we categorized 3D multicellular spheroid formation into two main categories: “3D cell-culture technology” and “multicellular cancer spheroid formation platform.” Three-dimensional cell culture refers to cells aggregated in a fluidic external cue or attached to an ECM-mimicking microenvironment. Compared to conventional 2D cell culture methods, it exhibits better tissue structure complexity and maintains numerous types of cell interactions. The 3D cell culture can be achieved through two approaches: anchorage-independent and anchorage-type approaches. The former allows effective nutrient uptake through high surface, and the latter enables stable cell growth in ECM-mimicking biomaterials. To fabricate real tumors, multicellular spheroids are adopted in the culture technology and platforms are employed to evaluate anticancer drug efficiency.

With the advent of 3D multicellular screening platforms, pharmacokinetic analysis of the MCTs can be realized. These platforms offer biologically similar in vivo structures of a solid tumor that can be conjugated to investigate complex mechanisms of tumor response to drugs that are not compatible with the 2D culture platform. Furthermore, they could be economically and ethically superior to 2D platforms as they simultaneously enable screening in bulk and reduce the frequency of animal model usage. Advancements in material science and drug-screening methods facilitate in-depth examination of the 3D culture platform. It is predicted that in the future, advanced biosensing tools will be developed to better evaluate drug efficacy and toxicity. Moreover, various applications are expected to be available for drug discovery, oncogenesis research, tissue engineering, and cell physiology, and to augment biomedical industry productivity.

Through the development of MCT generation platform and biosensing techniques, it enables accurate simulation of physiological response of tumor cells, allowing for in-depth analysis of oncology study. With highly simulated cancer models, it is accessible to track cancer cells and biological changes that regulate the behavior of tumors and responsiveness to clinical drugs. Recently, patient-derived cancer organoid (PDO) models, which are elicited from patient tumor tissues, have been an alternative to in vitro models in the biomedical field. Combining three-dimensional cell culture techniques and PDO models, patient-specific drug therapy will be secured and allow safe and efficient treatment, increasing the possibility of survival in the future. 

## Figures and Tables

**Table 1:** Spheroid formation technologies and platforms for cancer cells.

Cell Line	Culture Method	Substrate Type	Ref.
MCF-7, MDA-MB231	Hanging drop	-	[28]
BT474	Hanging drop	-	[29]
MCF-7	Hanging drop	Poly(N-isopropylacrylamide) (p(NIPA))	[30]
MDA-MB-231	Rotary cell culture system	-	[31]
HGC-27	Rotary cell culture system	-	[32]
HeLa, MCF-7, HUVECs	Porous scaffold	PDMS/CMC/PEDOT/Pt composites	[33]
PC3	Porous scaffold	Cellulose nanocrystals/poly(oligoethylene glycol methacrylate)	[34]
DU 145, A549, MCF-7, MDA-MB-231	Fibrous scaffold	Core–shell silk fibroin/rice paper	[35]
LNCaP	Gel-based scaffold	bQ13 (Ac-QQKFQFQFEQEQQ-Am) peptide	[36]
MCF-7, A549, A2780, P19, Panc02, UN-KC-6141	Hydrogel-based platform	Poly dimethyl siloxane (PDMS) derivatives (acoustic devices)	[37]
MCF-7	Hydrogel-based platform	ClO^−^/SCN^−^/carboxymethyl cellulose	[38]
NIH3T3, HepG2, HUVECs	Hydrogel-based platform	PEG-SH/Gela-SH/Gly-Tyr/D-PBS/HRP	[39]
A549, T24, Huh-7	Microwell-based platform	Polystyrene slides/PDMS	[40]
A549, MG-63, HLFs	Microwell-based hydrogel platform	N-isopropylacrylamide (NIPAM)	[41]
HepG2, HEK 293T	Microarray-based platform	Droplet microarray slides (DMA)	[42]
HUVEC, MCF-7	Matrix-based platform	Poly dimethyl siloxane (PDMS)/collagen	[43]
PANC-1, PS-1, HMEC	Matrix-based platform	Polyurethane/fibronectin (FN)/collagen I (COL)	[44]

**Table 2:** Drug-screening methods for multicellular cancer spheroids.

Cell Line	Anticancer Drug	Screening Tools	Ref.
MCF-7, HeLa, Caco-2	5-fluorouracil, cetuximab, panitumumab	Immunofluorescence	[115]
HT-29	Perifosine	Immunofluorescence	[116]
HT-29	Carboxyl-modified polystyrene nanoparticle	Immunofluorescence	[117]
Human breast cancer cell line (MCF-7 and MDA-MB-231)	Copper(II)-tropolone complex	Cell viability assay	[118]
MCF7, MDA-MB-231, SKBR3, MCF12A	Preussin	Cell viability assay	[119]
MCF-7	PLGA-MnO2 nanoparticles	Cell viability assay	[120]
HCT-116	Doxorubicin	Electrical impedance spectroscopy (EIS)	[121]
Human neuroblastoma and glioblastoma (SH-SY5Y, U-87MG)	Curcumin	Electrochemical detection (Cyclic voltammetry, Differential Pulse Voltammetry)	[122]
HCT-116	Immersion media (glycerol and ScaleView-A2)	Optical coherence tomography	[123]
MCF7	Paclitaxel	Optical coherence tomography	[110]

## Data Availability

Not applicable. No new data were created or analyzed in this study.
